# A role for aquaporin-4 during induction of form deprivation myopia in chick

**Published:** 2008-02-08

**Authors:** Melinda J Goodyear, Barbara M Junghans, Loretta Giummarra, Melanie J Murphy, David P Crewther, Sheila G Crewther

**Affiliations:** 1 School of Psychological Science, La Trobe University, Melbourne, Australia; 2 School of Optometry and Vision Science, University of New South Wales, Sydney, Australia; 3 Brain Sciences Institute, Swinburne University of Technology, Melbourne, Australia

## Abstract

**Purpose:**

Aquaporins (AQP) form a family of specialized water channels known to transport water across cell membranes and reduce osmotic gradients. The isoform AQP4 is highly expressed in the astroglia of the brain and Müller cells in the retina. In the brain, AQP4 play a role in the control of cerebral edema by shunting excess fluid into blood vessels and by upregulating during conditions of hyperosmolarity. Thus, on the basis of the hyperosmolarity seen across the retina and choroid of hatchling chickens made myopic by form deprivation (FD), we predicted an upregulation of retinal AQP4 expression during induction of myopia.

**Methods:**

Two-day-old hatchling chicks were monocularly form-deprived for 48, 72, or 96 h, and then after biometric assessment, the eyes of these animals and the normal controls of the same age were enucleated. Retinal tissue was prepared either for western blot analysis to show the presence of the AQP4 protein in the chick retina or for immunolocalization using polyclonal AQP4 antibodies to determine regional distribution and intensity of labeling during the induction of form deprivation myopia (FDM).

**Results:**

As expected, ultrasonography demonstrated that all post hatchling eyes showed rapid elongation with occluded eyes elongating faster than fellow eyes or normal controls and becoming progressively more myopic with the duration of visual deprivation. Western blot analyses revealed an approximately 30 kDa band immunoreactive for AQP4 protein and confirmed the presence of AQP4 in chicks. Immunohistochemical staining showed the greatest positive immunoreactivity for antibodies to AQP4 in the inner retina along the vitreoretinal interface, nerve fiber layer, ganglion cell layer, and inner plexiform layer in all animals. The control eyes showed relatively constant levels of AQP4 expression until day 5 after which the level appeared to decrease. By comparison, positive AQP4 immunoreactivity in the nerve fiber layer increased significantly over the first 48 h in form-deprived eyes and in fellow eyes and then decreased over the next 48 h but not to the level of expression in the normal untreated eyes.

**Conclusions:**

This is the first study to demonstrate the presence of AQP4 protein in the chick retina and to associate AQP4 expression in the inner retina with the initiation of form deprivation and the period of fastest axial elongation. This increased expression of AQP4 channels near the vitread border during the time of rapid growth suggests a role for AQP4 as a conduit for movement of retinal fluid into the vitreous in form-deprived chicks.

## Introduction

Homeostatic control of water transport in cells and tissues is critical for survival and has been the subject of much study, particularly following the discovery of aquaporins in the early 1990s [1]. Prior to this, theoretical models of water control predicted the need for specialized water channels in cells, but no evidence existed to demonstrate that such pores existed (see reviews [2-8]). Aquaporins (AQPs) are now recognized as serving an important role in the distribution of fluid and in the reduction of osmotic and hydrostatic gradients with 13 different mammalian aquaporin proteins having been described (AQP0-AQP12) [9]. Many have been linked to important physiologic roles in fluid transfer associated with osmolarity and hydrostatic balance in tissues such as the brain, kidney, gastrointestinal tract, lung, and skin (see reviews [9-12]). Aquaporins AQP0, 1, 3, 4, 5, and 9 have also been identified in rodent and human eyes [13-19], although, currently there are no reports of aquaporins in the chick retina. This raises the important question of how retinal AQPs interact in the exquisite control of water and fluid necessary to maintain the integrity of the optical and light-sensing function of the neurosensory aspect of the eye [20-22].

Roles for AQP1 and AQP0 in human ocular conditions such as Fuch’s dystrophy, bullous keratopathy [23], and cataract [24] have been suggested. Studies on transgenic mice also indicate functional roles for AQP0 in cataract development [25], and AQP1 and AQP5 have been associated with corneal hydration and transparency (see review by Verkman [26]). In addition, AQP1 has been shown to be associated with aqueous production and the maintenance of intraocular pressure [26]. Verkman has suggested that AQP4, which is richly distributed in the Müller cells of the murine retina [14,16,17], may play a role in retinal signal transduction [22]. Müller cells, like other glial cells, are well known for their role in homeostatic control of the neural microenvironment [27]. Given that AQP4 is highly expressed on the endfeet of astroglia and is associated with the control of cerebral edema by shunting the excess fluid into blood vessels in response to hyperosmolarity in the brain [7,28], we reasoned that AQP4s may also be associated with control of osmolarity and hydrostatic balance in the retina.

If AQP4 does indeed play such a role in osmotic and hydrostatic-related functions of the retina, we would expect to see changes in the AQP4 expression in Müller cells in any ocular disorder characterized by abnormalities of retinal hydration or changes in vitreal volume such as in refractive errors like myopia and hyperopia [29]. Indeed, 95% of human myopic refractive errors are correlated with changes in vitreous chamber depth [30], suggesting that refractive error development is likely to be associated with mechanisms that induce differences in fluid homeostasis in the eye.

The need to understand the mechanisms that control ocular growth and the development of refractive errors cannot be underestimated as the prevalence of myopia and the degree of severity is rapidly increasing, particularly in some Asian communities where almost 90% of 18 year olds are now myopic [31]. The public health impact of this is enormous as not only is optical correction required for quality of life, but the ever increasing numbers with higher degrees of myopia will lead to a higher incidence of uncorrectable low vision [32] or blindness [33]. This evidence for a greater prevalence of low and high degrees of myopia in recent times highlights the relationship between environmental issues and ocular growth.

The most common animal model used in the study of the development of refractive error is the hatchling chick where form deprivation (FD), produced by wearing a translucent occluder over an eye, induces morphological and gross volumetric changes as well as significant myopic refractive error within a few days [34-36]. These changes in ocular size appear to be effected by mechanisms local to the eye [37,38]. The vitreal enlargement associated with FD is usually accompanied by a concomitant but inversely related reduction in choroidal thickness [39], possibly implicating the rate of net transfer of fluid between the vitreous and choroid in the development of refractive error [40] and alterations in choriocapillaris permeability [41-43]. In all species studied to date, the normal functioning of the retina is characterized by a net transport of fluid across the retinal pigment epithelium (RPE) from retina to choroid [44]. This fluid transfer across the retina is facilitated by the Müller cell, which has been suggested [16,21,27,45] to be the principal route for rapid water transfer across the retina and responsible for the maintenance of the extracellular osmolarity during neuronal activity in the retina.

In the past, much research on the etiology of refractive errors and associated ocular growth has focused on mechanisms of scleral and choroidal growth as the primary agents of growth [29] rather than considering the growth of such ocular coats as a more passive resultant of the increase in the vitreous volume and hence the ocular volume [35,36,46]. Indeed, vitreal increases would be expected if form deprivation myopia (FDM) is characterized by hyperosmolarity as indicated by relatively large increases in sodium (Na^+^), chloride (Cl^-^), and potassium (K^+^) ions in the outer retina and Na^+^ and Cl^-^ in the inner retina of form-deprived eyes compared to their fellow eyes that did not undergo FD. Such increases in ion abundances have been shown to dissipate following occluder removal and during refractive normalization [36,46].

Thus, we aimed to investigate whether AQP4 channel expression would correlate with axial elongation by comparing the effect of two to four days of ocular occlusion in hatchling chicks on the expression of AQP4 with hatchling chicks undergoing normal development at the same age. The four-day duration of occlusion was chosen for this examination as pilot studies indicate that this is the period of fastest ocular growth in the development of FDM.

## Methods

### Animals and rearing

Fifty-two hatchling white Leghorn-Australorp cross chicks were raised with unlimited food and water in a controlled environment on a 12 h light/12 h dark cycle (40 W incandescent light) and with the temperature maintained at 31–32 °C. Fifteen chicks were monocularly deprived by a translucent occluder, and their fellow eyes acted as controls for varying times from day 2 (48, 72, or 96 h). The translucent occluders were heat-formed hemispheres molded from translucent white styrene sheets and attached to the periocular feathers of the chicks with contact adhesive. Eleven pairs of eyes were enucleated after death and successfully prepared for immunological comparison: T=48 h (n=3), T=72 h (n=4), T=96 h (n=4). Thirty-two chicks were raised normally without any intervention at another time to serve as normal, age-matched controls for all points of occlusion from which 28 successfully prepared sets of eyes were available for immunological comparison (thus providing ‘normal’ controls for T=0 h [n=3], T=24 h [n=6], T=48 h [n=6], T=72 h [n=6], and T=96 h [n=7]). Eight eyes from another four chicks that were raised normally without intervention and both eyes of one chick that was raised under conditions of monocular deprivation were used for western blot analysis.

**Figure 1:**
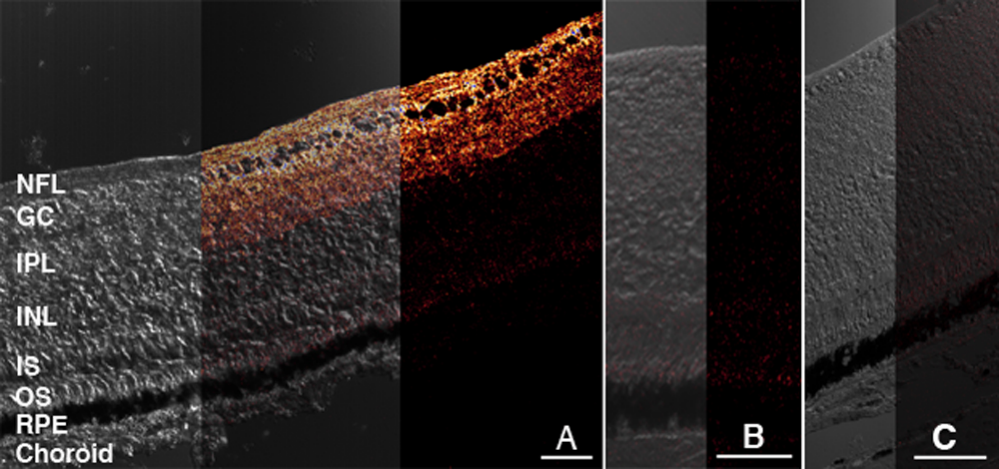
AQP4 immunolabeling of the normal chick retina plus negative controls. **A:** Confocal micrograph (40X magnification) with red AQP4 immunolabeling (to the right) of a transverse section of a normal chick retina merged (in middle) with the accompanying differential interference contrast image (to the left) is shown. The layers of the retina are indicated: NFL nerve fiber layer, GC ganglion cell layer, IPL inner plexiform layer, INL inner nuclear layer, IS inner segments of photoreceptors, OS outer segments of photoreceptors, RPE retinal pigment epithelium. It can be seen that labeling is most intense within the NFL, GC and IPL layers. Only very weak label is visible in the INL and photoreceptor region. **B**: Negative control with the primary antibody omitted indicates very weak red non-specific labeling (to the right) merged with the accompanying differential interference contrast image (to the left). **C**: Tissue pre-incubated with purified peptide before AQP4 labeling is shown indicating that blocking of AQP4 sites with the purified peptide successfully prevents binding of the introduced AQP4 antibodies and apparent red immunolabel. Bars=50 μm.

### Biometric analysis

At the end of the period of FD, biometrics were conducted using retinoscopy to determine refractive error and ultrasonography (Ophthasonic A Scan III; Teknar Inc., St Louis, MO; 6 MHz, 0.01 mm accuracy) to determine axial length (anterior cornea to internal limiting membrane of retina). All experimental procedures were performed under general anesthesia induced by intra-muscular injection of a mixture of ketamine (45 mg/kg) and xylazine (4.5 mg/kg). Animals were decapitated under general anesthesia, and the eyes were immediately prepared for western blotting and immunohistochemical labeling. Animals were raised and killed in accordance with La Trobe University Institutional Ethics and the regulations of the National Health and Medical Research Council of Australia and conformed to the United States NIH document ‘Guiding Principles in the Care and Use of Animals 1996’.

### Western blot analysis

As antibodies against chicken AQP4 were not commercially available, steps were taken to ensure the use of commercially available antibodies against rat AQP4 could be justified for use in chick. A homology between chicken and rat was calculated and found to be 75% to residues 249–323 of rat AQP4 (Homologene Blast comparison of *Rattus norvegicus* AQP4, Accession Number NP_036957.1, with *Gallus gallus* AQP4, Accession Number NP_001004765), and pilot studies using the Chemicon (Temecula, CA) polyclonal rabbit anti-rat AQP4 antibodies were performed, indicating sufficient conserved regions to conduct immunohistochemistry in the chick retina with an antibody to rat AQP4. The Chemicon antibody in use was raised against the glutathione-S-transferase (GST) fusion protein with residues 249–323 of rat AQP4 (SwissProt Accession Number P47863).

In addition, western blot analyses were performed. In a pilot study, retinal tissue from three normal chicks and stored brain tissue from four adult Hooded Wistar rats were used. Subsequently, retinal tissue was taken from both eyes of one normal chick and from both the form-deprived and the fellow eye of another chick. The extraction of proteins was conducted by homogenization of tissue in an ice-cold lysis buffer containing 20 mmol/l Tris-HCl (pH 7.5), 150 mmol/l NaCl, 1 mmol/l EDTA, 0.5% Triton X-100, 0.1% SDS, and protease inhibitors (Complete Protease Inhibitor Cocktail; Roche Molecular Biochemicals, Indianapolis, IN). The supernatant was collected and stored at −80 °C. Protein content was determined using a Bicinchoninic Acid Protein Assay Kit (Sigma-Aldrich, Saint Louis, MO).

For SDS–PAGE, the pilot study used rat tissue samples (50 µg/lane) that were diluted in Laemli buffer (Sigma-Aldrich) and resolved on a ready-made 12% Tris-HCl gel (Bio-Rad Laboratories, Gladesville, NSW, Australia) and chick samples (240 μg/lane) that were resolved on a 10% SDS polyacrylamide gel. Following electrophoresis, immunoblotting was performed by electrotransferring the protein to a nitrocellulose membrane (Bio-Rad Laboratories, (Hercules, CA) in a transfer buffer (25mM Tris, 192 mM glycine, 20% methanol (v/v), pH 8.3). The membrane with blotted proteins was blocked for 1 h at room temperature with Tris-buffered saline (TBS) containing 5% (w/v) skim milk powder, 1% (w/v) bovine serum albumin (BSA), and 0.1% Tween 20 followed by incubation overnight at 4 °C with polyclonal rabbit anti-rat AQP4 (Chemicon International) diluted 1:1000. After three washes at 10 mins each were done in TBS containing 0.1% Tween 20, the membrane was incubated with 1:2000 dilution goat anti-rabbit IgG (whole molecule) HRP-conjugated antibody (Sigma-Aldrich). Peroxidase activity was visualized using Lumiglo chemiluminescence reagents (Roche, Indianapolis, IN) and processed on autoradiographic film. Blots were reprobed with β-actin antibody (1:10,000; mouse monoclonal antibody; Sigma-Aldrich) to verify equal protein loading.

### Immunolabeling

Posterior eyecups fixed in 4% paraformaldehyde for 30 min were washed three times for 5 min each in phosphate buffered saline (PBS; pH 7.4) and incubated with 30% sucrose overnight at 4 °C for cryoprotection. Prior to sectioning, eyes were embedded in an OCT. compound (ProSciTech, Thuringowa, QLD, Australia), frozen, and stored at −20 °C. Sections of the posterior eye cup (10 μm) were cut on a Leica CM 1850 cryostat (Leica Microsystems, Heidelberger, Germany) then thaw-mounted and dried on StarFrost positively charged slides (ProSciTech) before being stored at −20 °C.

Sections were incubated for 1 h in a 3% goat serum blocking solution with Triton X-100 (AG Scientific, San Diego, CA) in PBS at room temperature followed by primary antibody application overnight at 4 °C with 0.4 µg/ml affinity-purified polyclonal rabbit anti-rat AQP-4 antibody (#AB3594; Chemicon). Antibody dilutions were previously tested using a range of dilutions with 0.4 µg/ml concentration chosen as the optimal dilution. Slides were washed three times in PBS before incubation for 1 h at room temperature with the fluorescent secondary antibody, Alexa 596-conjugated goat anti-rabbit IgG (Molecular Probes, Eugene, OR), diluted to 5 µg/ml in blocking solution. Negative control sections were processed with the primary antibody omitted from one section per slide as well as with the antibody pre-incubated with purified peptide supplied by Chemicon according to the manufacturer’s instructions. All sections were washed and coverslipped with Gel/Mount mounting medium (ProSciTech). The immunolabeling for the age-matched control chicks was run separately from the labeling of experimental chick tissue, but the same batch of antibodies was used. To ensure parity of labeling intensity between batches, slides with tissue from five of the experimental chicks were included with the age-matched normal tissue to compare intensity of labeling across batches and permit normalization of the intensity of control tissue labeling.

**Table 1:** Mean ocular axial length and refractive error after varying periods of visual form deprivation in the chick.

**Time (h)**	**Number of eyes in each condition**	**Eye**	**Axial length (mm)**	**Mean refraction (D)**
0	6	Normal control	8.29±0.05	0.97±0.35
24	6	Normal control	8.57±0.09	-0.17±0.55
48	3	Deprived eye	9.08±0.25	-6.0±2.08
	3	Fellow non-deprived eye	8.64±0.06	0.8±1.20
	6	Normal control	8.59±0.14	0.60±0.74
72	4	Deprived eye	9.32±0.05	-7.6±1.04
	4	Fellow non-deprived eye	8.85±0.03	3.5±0.65
	7	Normal control	8.77±0.10	0.11±0.55
96	4	Deprived eye	9.52±0.07	-13.3±1.85
	4	Fellow non-deprived eye	8.75±0.05	3.0±0.41
	6	Normal control	8.77±0.06	-0.10±0.52

Descriptive biometric statistics showing numbers of animals, eye treatment, mean axial length for each group in mm and mean refraction in dioptres (D) at time (hours post initiation of occlusion period) of enucleation.

### Image analysis

Labeled sections were observed under a Leica TCS-SP2 confocal laser scanning microscope (Leica Microsystems, Heidelberger, Germany). Initially, all immunolabeled sections of transverse retinal sections were examined qualitatively. However, once a similar pattern of positive immunoreactivity within both central and peripheral retinal regions had been established for each section, only images showing regional distribution and change in intensity of labeling at the posterior pole were captured and examined using the Leica software set at 4X line averaging without full frame averaging.

Images were first obtained at 40X magnification using pre-determined photomultiplier (PMT) settings (600 V, 650 V, 700 V, and 750 V) to determine the optimal PMT setting that would yield adequate images for comparison of labeling between FD eyes and fellow or control eyes. In all images, only the inner retina was specifically labeled for AQP4 (see Figure 1). Thus, further images were captured at 100X magnification and labeling intensity was analyzed using NIH ImageJ software (National Institutes of Health, Bethesda, MD) for the nerve fiber layer (NFL) and the inner plexiform layer (IPL) as confirmed by differential interference contrast microscopy (Figure 1A). The NFL and IPL were chosen to be measured in their labeling intensity as these layers of the retina showed the highest intensity of continuous specific labeling across all eccentricities of the retina. Mean luminance values (average brightness per pixel) were determined from brightness histograms as it is generally accepted that pixel intensity is proportional to chromagen concentration under certain conditions [47-49]. Luminance histograms were generated from manual tracings across the full width and thickness of the NFL or IPL for each image and from applying the ‘analyze – histogram’ tool in ImageJ to each area. Mean luminance and the number of pixels analyzed for each area traced was recorded. Manual tracing was made more manageable by subdividing the NFL of each image into four areas and the IPL into 10 areas, and the 4 or 10 independent mean luminance values per image were then treated as a nested variable. The luminance values were thus derived from an average selection of a total of 5,760 pixels for the NFL and 22,295 pixels for the IPL of each eye, which provided an acceptable degree of variance. No subtraction was made to allow for background labeling of either experimental, fellow, or control eyes as it was judged to be equivalent in all cases.

### Statistical analysis

The analysis of variance (ANOVA; Statview SAS Institute Inc. Cary, NC) was performed between occluded and fellow eyes to determine significant changes in labeling intensity according to visual status and time of occlusion. Bartlett’s Test of Sphericity indicated the appropriateness of parametric analysis. The multiple luminance measures for each animal were assigned as a compacted variable to provide a nested ANOVA. Post-hoc analyses were undertaken to determine the significance of the differences in refraction and axial length between deprived and fellow non-deprived eyes. Differences in labeling intensity between normal control eyes against fellow non-occluded eyes and occluded eyes were determined by one-way ANOVA with post-hoc comparisons.

**Figure 2:**
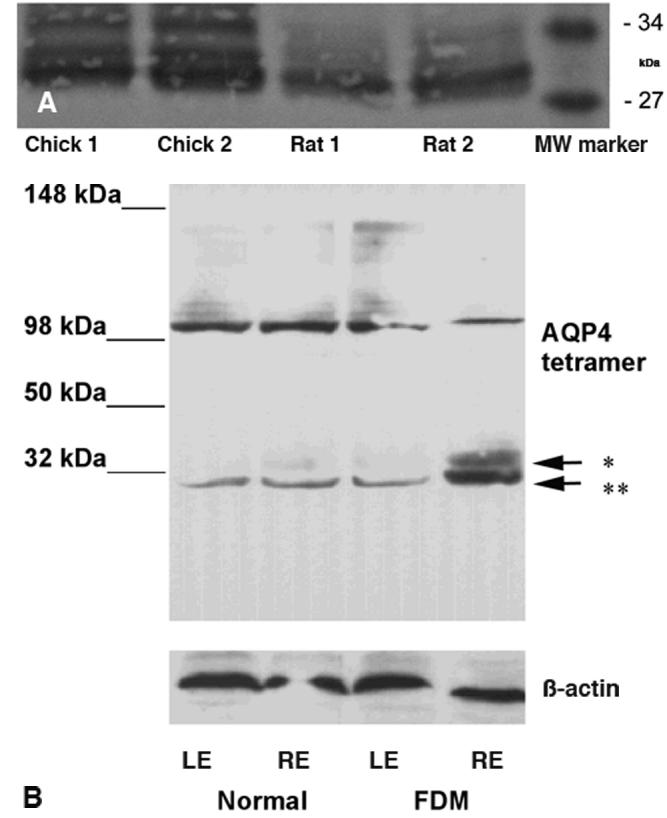
Western blot analyses of AQP4 in chick retina and rat brain. **A.** Western blots of retinal tissue from three normal chicks (lanes 1 and 2) and brain tissue from four Hooded Wistar rats (lanes 3 and 4) is displayed. Lane 5 is the molecular weight marker. **B **shows the western blot analyses of AQP4 antibody in chick retinae: tissue from left (LE) and right eyes (RE) of a normal control chick in lanes 1 and 2 alongside tissue from fellow non-deprived left eye (LE) and form-deprived right eye (RE) of an experimental chick in lanes 3 and 4. All eyes show a similar band around 30 kDa. The form-deprived eye appears to display strong bands for both isoforms of AQP4 at about 30 kDa (indicated by the double asterisk) and 32 kDa (indicated by a single asterisk). Note that a band is visible at about 100 kDa and may potentially relate to a tetramer of AQP4.

## Results

### Ocular growth and refractive status

All eyes continued to elongate during the week after hatching with the occluded eyes growing more rapidly (p<0.01) than their fellow non-occluded eyes, which showed no significant difference in the growth rate to the normal eyes (see Table 1). The rate of increase in axial length for normal control eyes and fellow non-occluded eyes appeared to be variable over the first six days, whereas the rate of growth in the experimental eyes was more rapid and consistent. The variability in growth rate between normal control eyes and fellow non-deprived eyes may be attributed to the fact that the raising of control chicks was during the summer while the experimental chicks were raised during winter as the hatchery does not provide temperature controlled conditions before and during delivery. This initial rearing appears to particularly affect their constitution in the winter. The occluded eyes developed myopic refractive errors (p<0.0001) proportional to the duration of form deprivation (see Table 1).

### Western blot

The pilot immunoblot analysis revealed that the immunoreactive protein was present in both the rat brain and chick retinal tissue samples (Figure 2A). The appearance of bands between 30 kDa and 32 kDa, which is the estimated size of the AQP4 molecule in other species [50], indicates that AQP4 is present in tissue from both the rat brain and the chicken retina. The subsequent immunoblot analysis of normal, form-deprived, and fellow non-deprived eyes of the chicks revealed that the immunoreactive protein was present in retinal tissue samples (Figure 2B). Again, the appearance of bands between 30 kDa and 32 kDa indicates that AQP4 is present in tissue from the chicken retina. The 30–32 kDa band from the form-deprived myopic eye revealed a stronger presence of protein than for either the fellow non-deprived or normal control eyes both of which demonstrated similar levels of protein.

**Figure 3:**
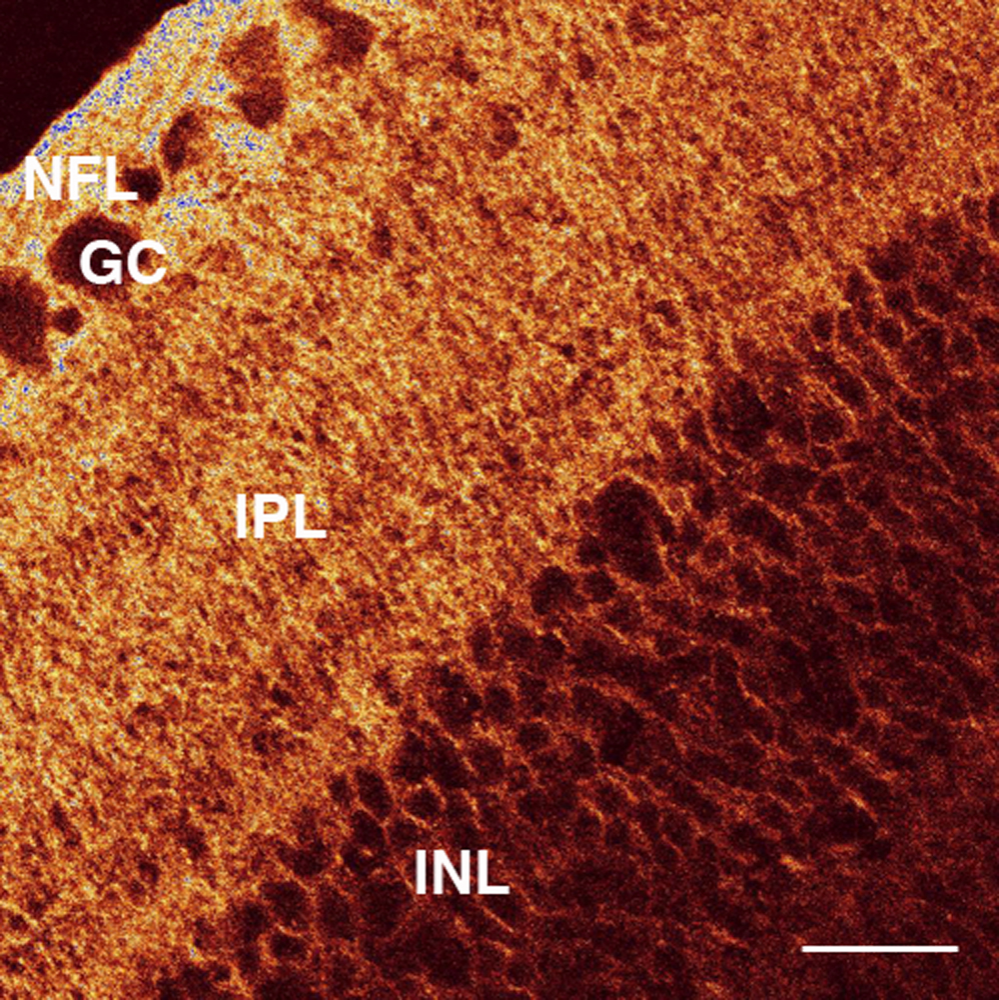
AQP4 labeling of the chick retina. A confocal micrograph of a transverse section of the retina from a form-deprived eye after 72 h of occluder wear is shown. There is strong labeling in the nerve fiber layer (NFL), ganglion cell (GC) layer, and inner plexiform layer (IPL) but very weak labeling in the inner nuclear layer (INL). Note the branching brush-like appearance of the pattern of AQP4 expression and also the appearance of several sublaminae in the IPL, which is similar to the previously described morphology of the Müller cell [51]. Bar is 20 µm.

### Immunoreactivity in normal eyes

AQP4 expression showed a regionalized distribution across the transverse retina in all eyes. The most intense AQP4 immunoreactivity was always exhibited in the innermost portions of the retina along the vitreoretinal interface, the NFL, ganglion cell (GC) layer, and the IPL (Figure 1A). The appearance of the many interweaving branching processes matched that of the morphology of the chick Müller cell within the IPL and GC (see Figure 3) as first illustrated in the chick by Cajal Golgi staining (reproduced subsequently in Dreher et al. [51] using 4F3 and 4H11 immunocytochemical labeling. This study also confirmed the appearance of the many interweaving branching processes by the overlay of the immunolabeling image on the differential interference contrast image. The strongly labeled filamentous processes appeared as tightly wrapped bundles around individual ganglion cells (Figure 1A). Several sublaminae outlined by the pattern of AQP4 labeling could be seen within the IPL.

AQP4 labeling was minimal within the inner nuclear layer, outer plexiform layer, outer nuclear layer, inner segments, outer limiting membrane, and outer segments (Figure 1A). The diffuse background labeling and oil droplet fluorescence were seen around the photoreceptors (Figure 1A). Similar intensity of non-specific labeling was seen in each of the negative control slides with the primary antibody omitted (Figure 1B). Similarly, the lack of labeling in the peptide blocked slides confirms the specificity of the AQP4 antibody (Figure 1C).

**Figure 4:**
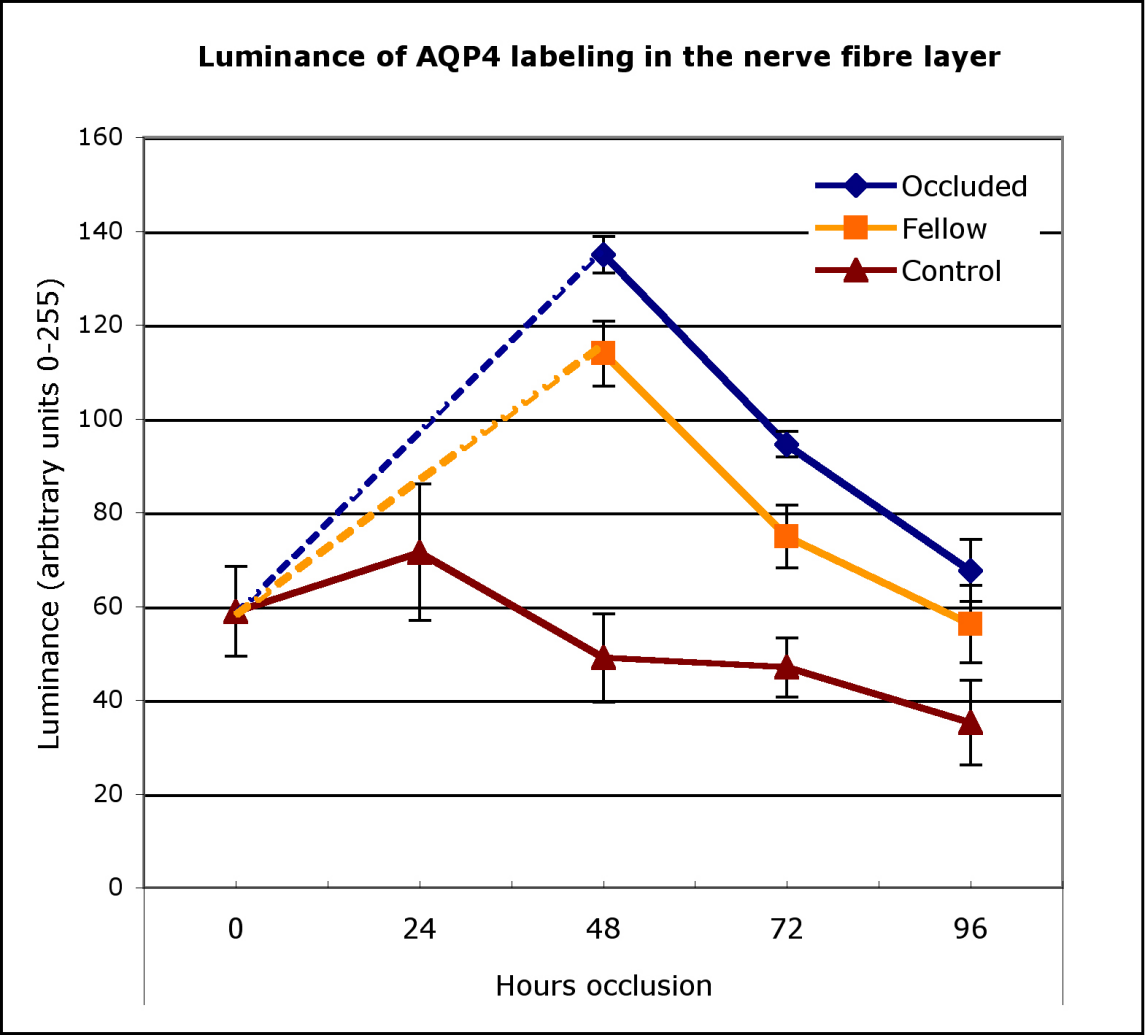
Intensity of AQP4 antibody labeling in the nerve fiber layer of chick. The mean relative fluorescence (on an arbitrary scale of 0 to 255) of AQP4/Alexa 596 conjugated antibodies expressed in the nerve fiber layer of the retinae of age-matched normal control eyes (triangles) and both non-deprived eyes (squares) and fellow eyes that experienced form deprivation (diamonds) is plotted against time from initiation of occlusion of the experimental eye. Note that initially intensity of AQP4 labeling is slightly raised in normal eyes and falls with time and increase in eye size. In contrast, occlusion initially causes significant elevation of labeling intensity particularly in the form-deprived eye, but also in the non-occluded fellow eye, which falls with duration of form-deprivation to almost reach control levels after 4 days. Error bars are 1 standard error.

In the normal non-treated controls, the intensity of labeling in the NFL of the normal eyes remained steady until day 5 after which labeling decreased (Figure 4). The pattern and level of expression in the IPL in the control eyes was similar to that seen in the NFL over this period.

### Immunoreactivity during form deprivation

**Figure 5:**
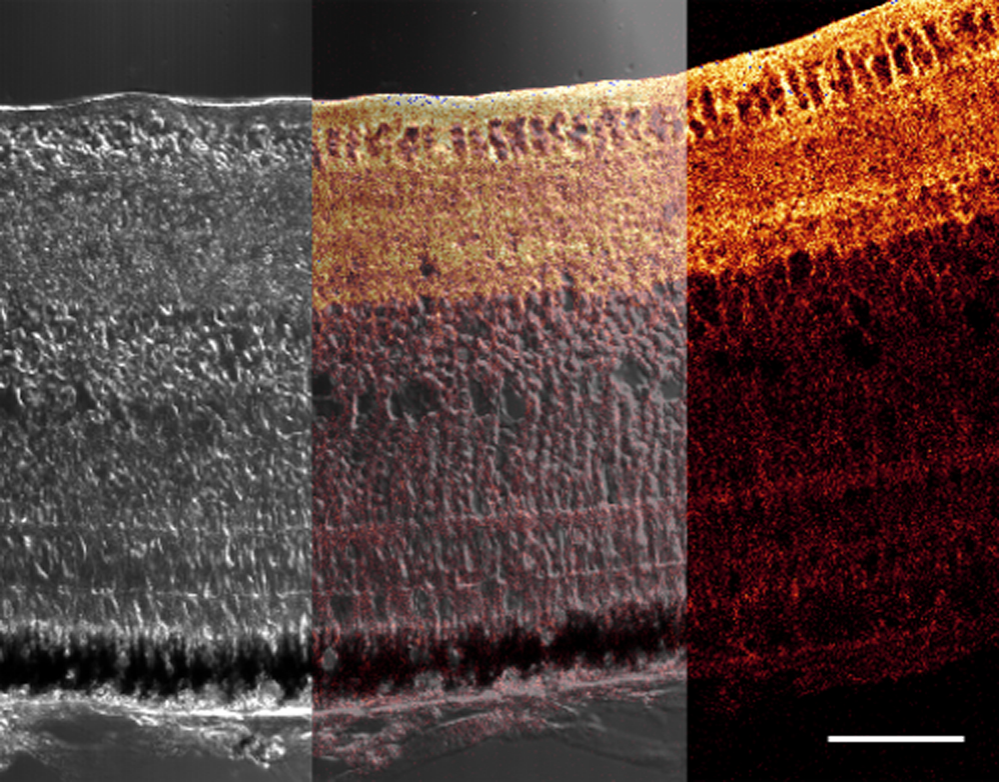
Confocal images of AQP4 labeling in the retina after 48 hours of occlusion. Composite confocal micrograph of a transverse section of the retina after 48 h of occluder wear is displayed. The left section demonstrates the differential interference contrast image and the right section illustrates the AQP4 immunofluorescent image in red. The middle section shows both images superimposed. Note that AQP4 labeling is predominant in the nerve fiber, ganglion cell and inner plexiform layers of the inner retina, just as demonstrated by the normal eye (see Figure 1). However, the intensity of labeling is greater along the vitreal border in the form-deprived eye than in a normal eye. Bar is 50 μm.

A similar regional distribution of AQP4 labeling as seen in normal control eyes was found for both the occluded and fellow non-occluded eyes (Figure 5). The main effects for the changes in intensity of NFL labeling (luminance) for refractive error [F(1, 82)=11.17, p=0.0012] and for the duration of occlusion [F(2, 82)=46.080, p<0.0001] were demonstrated. In particular, both occluded and their fellow eyes labeled far more strongly than the normal control eyes at 48 h [F(2, 11)=14.50, p=0.002], exhibiting at least double the luminance. There were also significant differences (p<0.01) in the luminance of AQP4 NFL labeling between deprived and fellow non-deprived eyes at 48 h of occlusion (Figure 4) with the occluded eye showing greater immunoreactivity in most cases. These significant differences between deprived and non-deprived eyes were also present at 72 h [F(2, 13)=7.51, p=0.009] but became insignificant by 96 h. Although there was a trend toward upregulation of AQP4 labeling in the IPL in FD eyes as opposed to fellow eyes, this was not significant, indicating that the majority of upregulation of AQP4 was predominantly at the vitreal border.

## Discussion

This study is the first to use western blots to demonstrate the presence of AQP4 protein in chick retina. The study also describes the distribution of AQP4 expression in the neonatal retina of normal untreated chicks and describes a fall in AQP4 immunopositivity in the nerve fiber layer and the inner plexiform after postnatal day 5 as the daily rate of ocular growth falls. By comparison, the more rapid axial elongation after the initiation of occlusion of experimental eyes was accompanied by a pronounced upregulation of AQP4 expression within two days. As the rate of experimental growth declined with the duration of occlusion, a decreasing level of immunopositivity to AQP4 was also seen. Fellow non-deprived eyes demonstrated a less pronounced and variable pattern of axial growth. A comparison of the absolute axial length between normal control eyes and fellow non-deprived eyes is complicated by the rearing of control chicks during summer rather than winter when the experimental chicks were raised, resulting in heavier overall weight for the control chicks. The pattern of upregulation of AQP4expression in fellow eyes was similar to that described in the experimental eyes though not as pronounced. Thus, these results are the first to suggest that the expression of AQP4 is functionally associated with the development of axial refractive errors.

The localization of AQP4 seen predominantly in the innermost layers in chick retina bears striking similarity to that previously described in the rat retina [7,16,21] and matches the known morphology of the chick Müller cell [51,52]. Such a distribution close to the inner limiting membrane and vitread surface in young chicks would be expected to facilitate movements of water between the inner retina and vitreous [7,14,27,53,54] and to enable the rapid increases in ocular elongation seen during normal postnatal growth and the induction of myopic refractive errors. The appearance of sublaminae in the AQP4 labeling of the chick IPL concurs with the morphological Müller cell study of Dreher et al. [51] who speculated that such lamination reflects the presence of horizontal processes that might be involved in K^+^ uptake from the IPL. The IPL of the retina is the principal site of K^+^ release during the light-evoked response and the site from which K^+^ is known to be siphoned to areas of lower concentration such as the subretinal space (SRS) and the astrocytes surrounding the retinal blood vessels [21]. In the case of animals such as the chick and guinea pig that lack an inner retinal vasculature [55], K^+^ is thought to be deposited into the vitreous [21,56,57]. The absence of a retinal vasculature to act as a ‘fluid sink’ in these species could account for the rapid vitreal elongation seen in both species in response to form deprivation.

The decrease in AQP4 expression five days post-hatching in normal eyes is not surprising if, as we have postulated, AQP4 expression on the vitreal border is an indication of resultant fluid dispersion in ocular growth. The greatest increase in ocular volume and growth in chick as in monkey and humans [58] is immediately after birth, then ocular volume and growth slows down thereafter (See Table 1 and note largest changes in axial growth occur early). Thus, a decrease in the intensity of AQP4 labeling with days post hatching would be expected. Similarly, in the rat eye, Wurm et al. [57] have reported an increase in AQP4 expression in the plexiform layers at the inner limiting membrane and around blood vessels between eye opening at postnatal day (P) 15 and adulthood. However, by comparison to our results, Wurm et al. [57] did not note any significant changes in expression with visual deprivation between P18 and P20. A positive more extended developmental profile for AQP4 expression has also been described in the rat brain [59], which continues to increase in volume for a longer time after birth than the eye of chick, which is required to be functional at birth and is hence closer to adult proportions at an earlier stage.

The rapid abnormal elongation induced by occlusion is known to be accompanied by an increase in the amount of vitreal sol [60] and is supportive of a hypothesis that a reduction in the normal volume of fluid moves across the retina to the choroid [53]. Second, as AQP4 channels are known to act as bidirectional water channels on glia cells in the cerebral cortex in the presence of edema or changes in osmoregulation [61], it is not surprising that we have seen an associated, relative increase in the expression of AQP4 on the vitreal endfeet of Müller cells in the eyes experiencing occlusion compared to fellow eyes and normal control eyes. Such a bidirectional fluid flow (either from the vitreous to the retina and choroid or the reverse from the retina to the vitreous) would be expected if the AQP4s on Müller cells are to play a role in the retinal shunting of fluid between areas of change in osmoregulatory homeostasis as we have previously described in form derivation myopia [36,46].

The possible involvement of aquaporins as mediator of the water flux associated with K^+^ transfer was originally suggested by Nagelhus and his coworkers [21,62], who demonstrated the polarized colocalization of Kir4.1 potassium channels and AQP4 on Müller cell processes facing the vitreous and blood vessels in the rat retina [22]. Müller cells have previously been shown to play a role in the control of the neuronal microenvironment of the outer retina by regulating K^+^ concentration between the SRS and the vitreous during light-induced neuronal activation [27]. Such a role for the Müller cells in FD would support our earlier reports of changes in K^+^ [46,63,64], Na^+^, and Cl^-^ abundances [36,64,65] in the young chick retina and choroid during the recovery from form deprivation-induced myopia. Nagelhus and coworkers suggested that AQP4 might facilitate the rapid directing of water “to select extracellular compartments while protecting others (the subretinal space) from inappropriate volume changes” [21]. Similar arguments for a physiologic role in ion and water homeostasis by AQP4 in the glia in the brain have also been proposed in rat [7,10,62,66] and chick [28]. Furthermore, AQP4 knockout mice have been used to determine the potentially important relationship between the rapid movement of water during retinal signal transduction and AQP4 expression on Müller cell membranes [22].

In summary, we conclude that the presence of AQP4 on chick Müller cells is not simply a phenotypic variation without physiologic significance [67]. Rather, our results suggest a specific role for these specialized water channels during ocular growth in general and also during the development of refractive errors that follow visually mediated change in the axial dimension. Thus, the perturbations to AQP4 expression may be regarded as a consequence of changes to retinal signal transduction during reduced visual experience under an occluder. Such a change in photoreceptor and inner retinal activity requires Müller cells to reestablish ion homeostasis, resulting in ocular enlargement.
